# Key differences between olfactory ensheathing cells and Schwann cells regarding phagocytosis of necrotic cells: implications for transplantation therapies

**DOI:** 10.1038/s41598-020-75850-8

**Published:** 2020-11-03

**Authors:** L. Nazareth, T. B. Shelper, A. Chacko, S. Basu, A. Delbaz, J. Y. P. Lee, M. Chen, J. A. St John, J. A. K. Ekberg

**Affiliations:** 1 Menzies Health Institute Queensland, Griffith University, Southport, QLD 4222 Australia; 2 Clem Jones Centre for Neurobiology and Stem Cell Research, Griffith University, Nathan, QLD 4111 Australia; 3 Griffith Institute for Drug Discovery, Griffith University, Nathan, QLD 4111 Australia

**Keywords:** Cellular neuroscience, Cell death

## Abstract

Transplantation of peripheral nervous system glia is being explored for treating neural injuries, in particular central nervous system injuries. These glia, olfactory ensheathing cells (OECs) and Schwann cells (SCs), are thought to aid regeneration by clearing necrotic cells, (necrotic bodies, NBs), as well as myelin debris. The mechanism by which the glia phagocytose and traffic NBs are not understood. Here, we show that OECs and SCs recognize phosphatidylserine on NBs, followed by engulfment and trafficking to endosomes and lysosomes. We also showed that both glia can phagocytose and process myelin debris. We compared the time-course of glial phagocytosis (of both NBs and myelin) to that of macrophages. Internalization and trafficking were considerably slower in glia than in macrophages, and OECs were more efficient phagocytes than SCs. The two glial types also differed regarding their cytokine responses after NB challenge. SCs produced low amounts of the pro-inflammatory cytokine TNF-α while OECs did not produce detectable TNF-α. Thus, OECs have a higher capacity than SCs for phagocytosis and trafficking, whilst producing lower amounts of pro-inflammatory cytokines. These findings suggest that OEC transplantation into the injured nervous system may lead to better outcomes than SC transplantation.

## Introduction

Injuries to the central nervous system (CNS), such as traumatic brain injury and spinal cord injury, can be devastating due to the inability of the CNS to effectively regenerate. One of the contributing factors that limit the regenerative capacity is the presence of cell debris and necrotic cells within the injury site. After the initial trauma, cell death occurs via necrosis or apoptosis^1,2^, with the dying cells, in particular necrotic cells, releasing danger-associated molecular patterns (DAMPs) that produce an inflammatory response. This in turn activates and recruits resident microglia and astrocytes, as well as infiltrating immune cells^3^. While microglia are competent phagocytes, they cannot efficiently clear all the debris arising from a severe injury with both cellular and myelin debris observed years after CNS injury^4^. Astrocytes, in turn, are less efficient phagocytes than microglia^5^. The long-term presence of debris is thought to contribute to the lack of nerve regeneration after CNS trauma^6^.

In peripheral nerves, however, the scenario is vastly different; unless the damage is extensive, peripheral nerves can regenerate^7^. One peripheral nerve, the olfactory nerve, has particular ability to regenerate as the primary sensory neurons in this nerve are replaced throughout life. In this nerve, axonal debris is being constantly phagocytosed by the resident glia, olfactory ensheathing cells (OECs)^8^. After injury to the olfactory nerve, the OECs migrate to the injury site and rapidly phagocytose the debris^9–11^. In contrast to other regions of the nervous system, macrophages do not appear to be recruited to aid phagocytosis within the olfactory nerve fascicles^8,11^. We have recently shown that OECs express macrophage migration inhibitory factor (MIF) which repels macrophages from OECs^12^. It is also thought that OECs do not produce pro-inflammatory cytokines that would lead to recruitment of macrophages, however, this has not been clearly demonstrated^12,13^. Thus, the phagocytic clearance of cell debris in the olfactory nerve appears to be performed solely by OECs^10–12^. In other peripheral nerves, the resident glia, Schwann cells (SCs), respond to injuries by initially phagocytosing and clearing cell debris, and they also express pro-inflammatory cytokines that activate and recruit local and peripheral macrophages^7,14^. Thus, in contrast to OECs, SCs act together with macrophages to phagocytose cell debris.

Transplantation of OECs and SCs is being investigated as a potential therapy for repair of CNS injuries, in particular spinal cord injury, however, outcomes vary dramatically and the methods need to be improved^15,16^. The transplanted cells are thought to promote regeneration by secreting growth factors and guidance factors, by providing physical support, and by clearing debris arising from dead cells^13,15^. It has also been suggested that stimulation of the phagocytic activity to more rapidly clear the injury site of cell debris will further promote regeneration^13,17–21^. To date, it remains unknown how peripheral glia recognize, internalize and process necrotic targets. Understanding how these glia clear necrotic debris may reveal novel drug targets by which the glia can be stimulated to more efficiently clear debris. It has also been debated whether OECs or SCs are better at promoting neural regeneration. In fact, both these cell types are thought to favourably modulate the hostile, pro-inflammatory environment at the injury site after spinal cord injury, in particular by modulating reactive astrocytes^22–25^, but as OECs and SCs are phagocytic and can produce cytokines^9,24,26,27^, they may also release pro-inflammatory mediators that may adversely affect the injury site. Understanding how the two cell types respond to necrotic cells may reveal differences in their phagocytic capacity, as well as their potentially disadvantageous pro-inflammatory responses.

Cells undergo necrosis either in a programmed manner (necroptosis, ferroptosis and pyroptosis) on exposure to inflammatory mediators, or non-programmed necrosis after mechanical trauma, extreme heat or chemical treatment^28^. In both cases, necrotic targets, hereafter referred to as necrotic bodies (NBs), undergo swelling, membrane permeabilization and release of cellular contents. NBs also start to display specific molecules that phagocytic cells recognise, in particular phosphatidylserine (PS), on the plasma membrane^28–30^. Further, as cells regularly undergo apoptosis, clearing of apoptotic targets is a silent event without generation of immune response^31^. However, necrosis usually occurs as a part of injury or infection with a large amount of inflammatory mediators generated both by the dying cells and by surrounding cells^28^. In the current study, we investigated how OECs and SCs, recognise, respond to and phagocytose NBs, and compared their responses to those of macrophages, which are highly efficient phagocytes (commonly referred to as “professional phagocytes”). We further examined how glia responded to an inflammatory stimulus representative of an injury environment by secreting cytokines and compared the cytokine responses to those elicited by the presence of NBs. For successful repair, the transplanted glia would also need to phagocytose and clear myelin debris accumulated at the site of injury. Hence, we also investigated glial-mediated phagocytosis of myelin debris.

## Results

### Peripheral glia can internalize necrotic bodies

Peripheral nervous system glia are known to phagocytose various targets such as apoptotic cells, axonal debris and heat-killed bacteria both in vitro and in vivo, but phagocytosis of necrotic bodies (NBs) has not been characterized^10,18,32–36^. In the current study, we investigated whether two types of peripheral glia, olfactory ensheathing cells (OECs) and Schwann cells (SCs), could recognize and engulf NBs. We compared this activity in the glia to that of macrophages, which are efficient at recognizing and phagocytosing NBs^30,37–39^.

**Figure 1:**
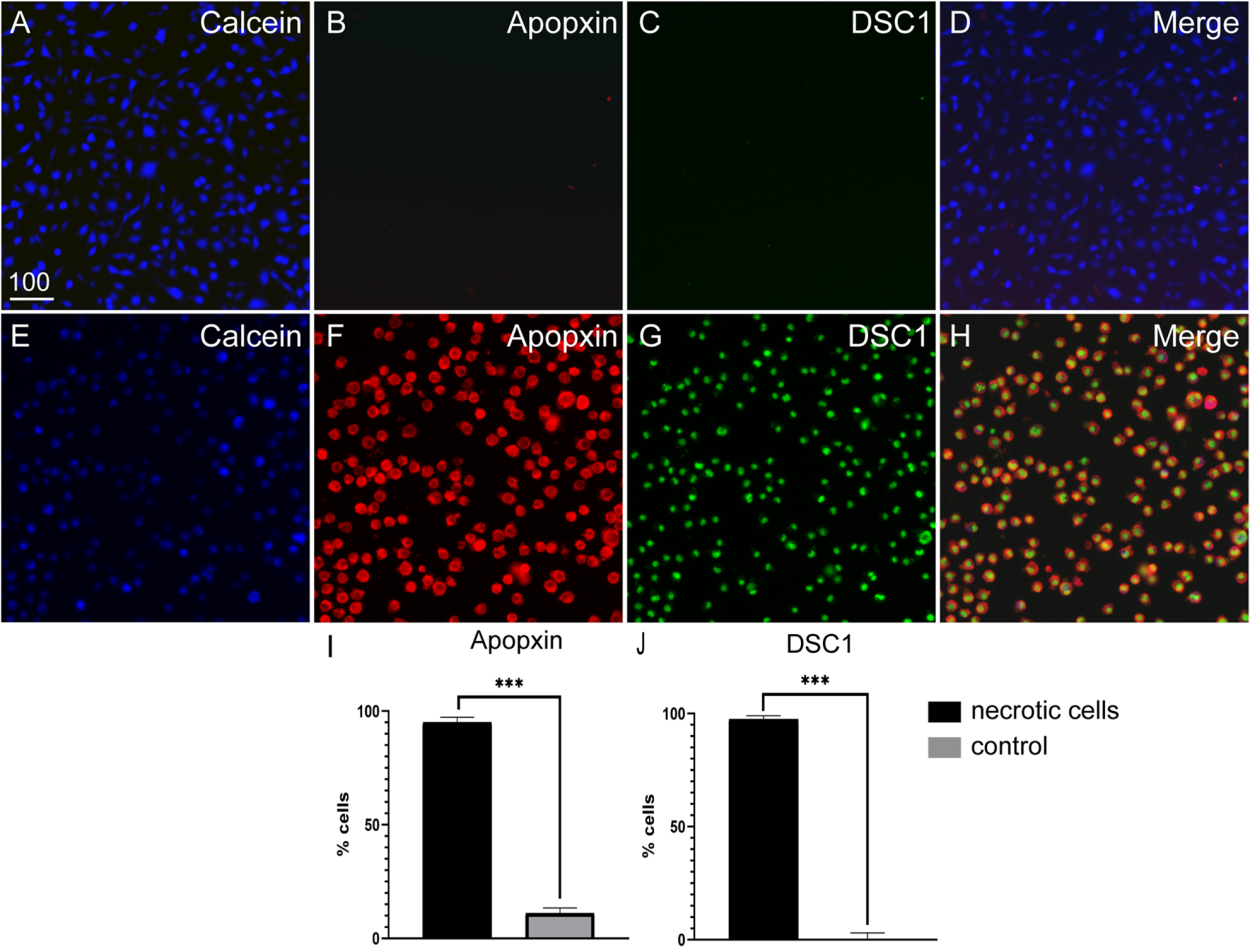
Heat exposure induces necrosis of McCoyB cells. The cells were either left untreated or induced to undergo necrosis by heat exposure at 55 °C for 30 min, after which cells were stained and transferred to 96-well plates for imaging. All cells were labelled with cytocalcein (live cell stain, blue), PS sensory dye (Apopxin, red) and membrane-impermeable DNA nuclear dye (DCS1, green). Example images of (**A**–**D**) untreated control cells and (**E**–**H**) heat-treated cells. (**I**) Percentages of cells displaying PS. (**J**) Percentages of dead cells (cells labelled with DSC1). PS- and DCS1-positive cells were automatically counted using Nikon Elements software. ***P ≤ 0.0001 (unpaired t-test with Welch’s correction). Data represents mean ± SEM (3 biological × 3 technical replicates of ~ 400 cells × 4 FOV). Scale bar: 100 µm.

To create NBs, we induced necrosis in a fibroblast cell line (mouse McCoy B cells) using heat treatment^30,37,38^. To determine the optimal incubation time for necrosis induction, McCoy B cells were heated to 55 °C for varying time periods (5, 10, 15, 20, 25 and 30 min) followed by staining with Trypan blue and DRAQ7 (which both are membrane-impermeable nuclear stains, thus staining necrotic/dead cells). Maximum cell death (100%) was observed at 30 min post heat exposure (supplementary Fig. 1). NBs are recognized by phagocytes as they display phosphatidylserine (PS) on the plasma membrane. We therefore then investigated whether the NBs displayed PS by staining with them with the PS-sensor dye Apopxin Red, along with DSC1 (another membrane impermeable DNA nuclear dye suitable for multicolor analysis). The NBs were readily labelled with both Apopxin Red and DSC1 (Fig. 1). We also immunolabelled the NBs with the fluorescently labelled PS-specific binding partner Annexin V-Alexa Fluor 647 and confirmed that the cells displayed PS (supplementary Fig. 1).

**Figure 2:**
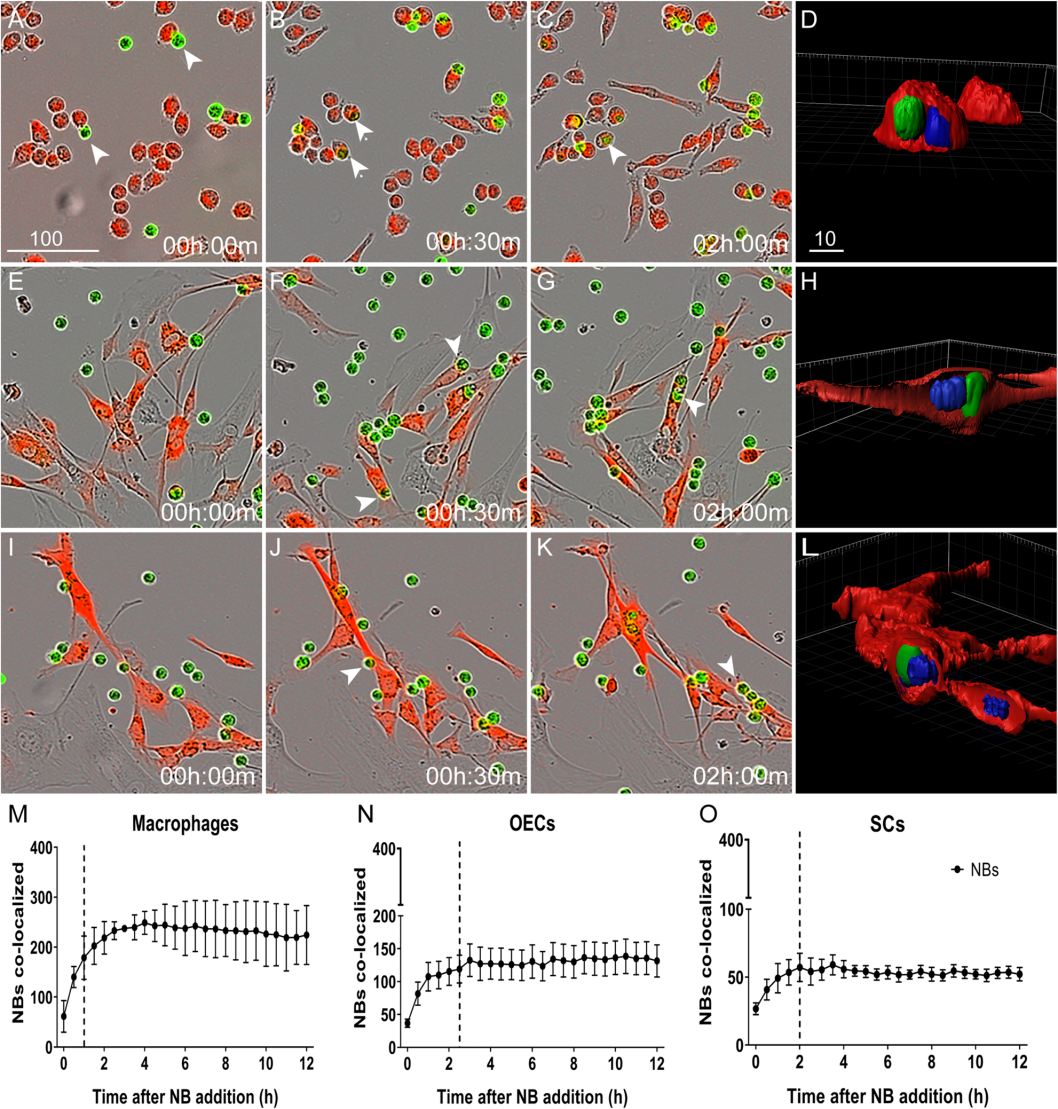
Engulfment of necrotic cells by macrophages, OECs and SCs. Example images of macrophages (J774A.1 cells) (**A**–**D**), OECs (**E**–**H**) and SCs (**I**–**L**) challenged with necrotic cells pre-labelled with cell tracker (CMFDA green), at a NB:live cell ratio of 4:1. OECs/SCs expressed DsRed; macrophages were labelled with CellTracker red dye. Arrows indicate cells establishing contact with NBs. Localisation of internalized NBs within cells was confirmed by confocal imaging and 3D rendering (**D**, **H**, **L**). (**M**–**O**) Graphical representation of the time-course for necrotic cell internalization by macrophages (**M**), OECs (**N**) and SCs (**O**). The Y-axes shows the number of NBs (green fluorescence) co-localizing with cells (red fluorescence) i.e. internalized NBs. The number of NBs co-localizing with cells at the various time-point were compared to background NB-cell co-localization levels (time zero). Stars shows the time-points at which the number of NBs co-localizing with cells were significantly different from background levels. *P ≤ 0.05, **P ≤ 0.01, ***P ≤ 0.001 (two-way ANOVA with Sidak’s multiple comparison test). Data represents mean ± SEM. n = 3 biological repeats × 3 technical replicates × 4 FOV/well (n = 300–400 cells/FOV). Scale bar: 100 µm in **A**–**C**, **E**–**G**, **I**-**K** and 10 µm in **D**, **H**, **L**).

To compare internalization of NBs between macrophages and glia, we used the J774A.1 macrophage cell line^40^ and primary OECs/SCs isolated from S100β-DsRed mice, in which all glia express the bright fluorescent protein DsRed^41^. We also verified that the DsRed-positive cells expressed the glial markers p75 neurotrophin receptor (p75ntr) and S100-β (supplementary Fig. 2). As comparison of internalization measurements are affected by cell proliferation, we compared the doubling time for OECs and SCs and the cell numbers present during the phagocytosis assays and found no difference between OECs and SCs (supplementary Figure 3). To assess internalization of NBs, we challenged the cells with NBs pre-labelled with CellTracker Green dye. The cells were imaged every 30 min, and co-localisation of necrotic targets with macrophages and glia was determined and quantified (Fig. 2). Necrotic targets rapidly bound to macrophages (Fig. 2A, arrows), with the number of NBs co-localizing with macrophages becoming significantly different from background (time zero) at 30 min (Fig. 2B,M) and reaching a peak at 1 h (Fig. 2B). OECs and SCs appeared to establish contact with necrotic cells at approximately 30 min post challenge (Fig. 2F (arrows),J). After 1.5 h for OECs and 1 h for SCs, respectively, the number of NBs became significantly different from background levels (time zero). NB internalization peaked at 2.5 h for OECs and at 2 h for SCs (Fig. 2N,O). The capacity for internalization differed considerably for the cell types, with macrophages internalising twice the amount of NBs in comparison to OECs, and OECs internalising twice the amount of NBs compared to SCs. To verify that the NBs were present inside the cells, we analysed the localisation of NBs using confocal microscopy. After 2 h, excess NBs were washed off, followed by fixation and confocal imaging; 3D rendering of the images confirmed that NBs were localised within the cells (Fig. 2D,H,L and supplementary videos 1–3).

### Necrotic bodies are processed in acidic intracellular glial organelles

**Figure 3:**
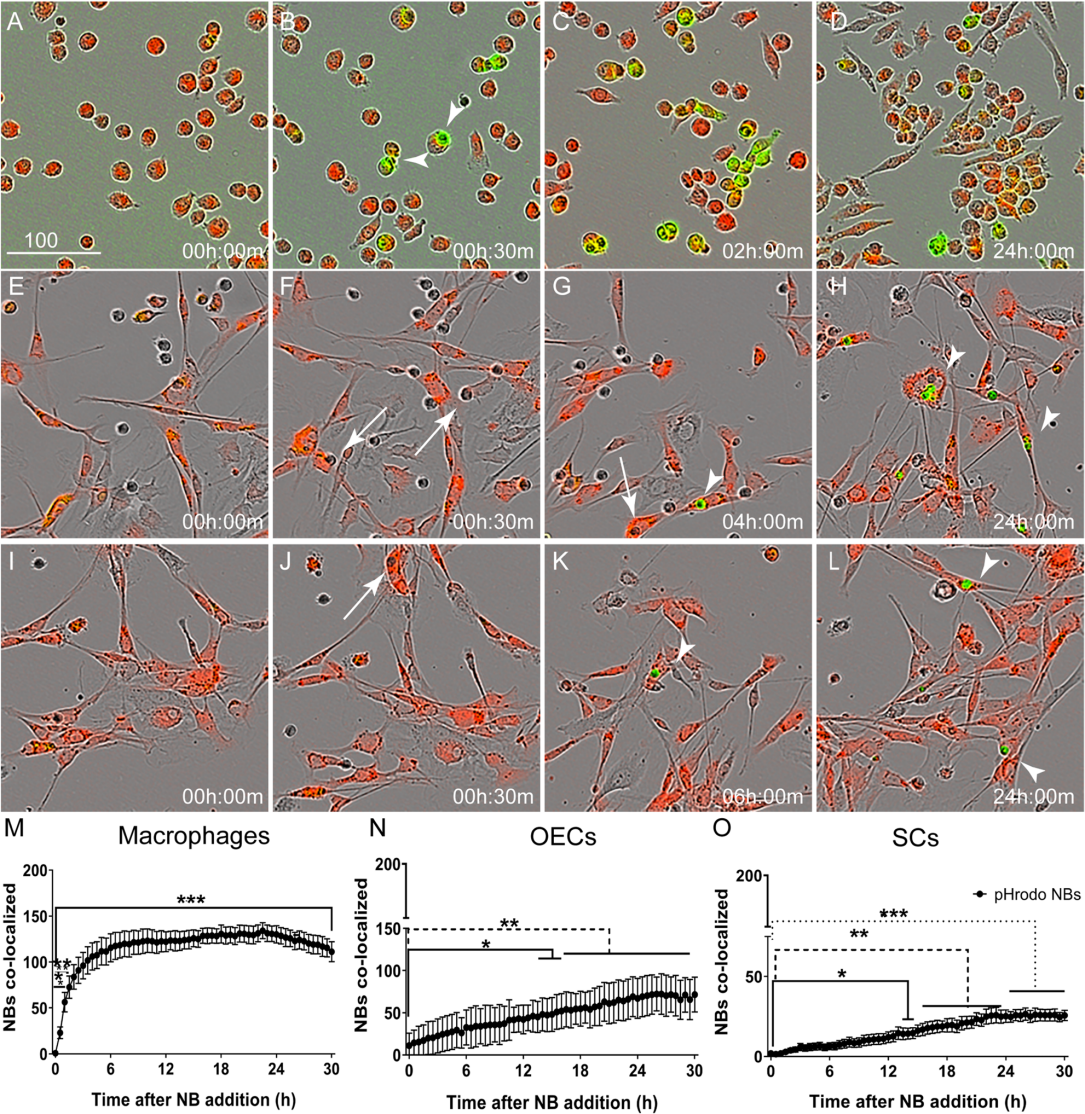
Trafficking of necrotic bodies to endosomes-lysosomes in macrophages and glia. (**A**–**L**) Example images of macrophages (J774A.1 cells) (**A**–**D**), OECs (**E**–**H**) and SCs (**I**–**L**) (red) with NBs inside endosomes/lysosomes (green, arrowheads). Necrotic cells were tagged with pHrodo STP dye (green) that only fluoresces in acidic pH (i.e. in intracellular endosomes/lysosomes); NBs not in endosomes/lysosomes do not fluoresce (examples shown by arrows in **F**–**H**). (**M**–**O**) Graphical representation of NB appearance within endosomes-lysosomes (pHrodo-tagged) between macrophages (**M**), OECs (**N**) and SCs (**O**). The Y-axis shows the number of NBs in endo/lysosomes (green fluorescence) co-localizing with cells (red fluorescence) i.e. internalized NBs. The number of pHrodo-tagged NBs co-localizing with cells at the various time-points were compared to background levels (time zero); stars shows time-points at which there was a significant difference from background *P ≤ 0.05, **P ≤ 0.01, ***P ≤ 0.0001 (two-way ANOVA with Sidak’s multiple comparison test). Data represents mean ± SEM. n = 3 biological repeats × 3 technical replicates × 4 FOV (n = 300–400 cells/FOV).

Phagocytosis is a complex process that requires phagocytes to recognize, engulf and efficiently degrade the internalized object^42^. Degradation occurs when the internalized object, surrounded by a vacuole (phagosome), fuses with acidic organelles (endosomes and lysosomes) causing the pH in the maturing phagosome to decrease, resulting in degradation of the cargo^43^. Hence, we next investigated whether NBs were trafficked to acidic organelles in glia, and studied the time-course for this process. NBs were labelled with the pH-sensitive dye pHrodo Green STP Ester dye (pHrodo STP), which fluoresces only at acidic pH (below pH 6). The tagged NBs were added to macrophages, OECs and SCs and imaged as described above. The cargo was internalized into acidic cellular compartment first by macrophages, followed by OECs and then SCs; the peak at which maximal amount of cargo was inside these organelles also occurred in this order. Green fluorescence was detected in macrophages 30 min after addition of NBs, and after 2 h, most macrophages exhibited green fluorescence (Fig. 3A–D). In OECs, fluorescing necrotic cargo was first detected at 2–4 h (Fig. 3E–H) and in SCs at 4–6 h (Fig. 3I–L) post NB addition (see also graphical representation of the time-course in Fig. 3M–O). The amount of fluorescent cargo in macrophages peaked at 2.5 h (Fig. 3M), in OECs at 23.5 h (Fig. 3N) and in SCs at 22 h (Fig. 3O). We also determined the total capacity for internalization into acidic organelles over time by measuring the area under the curve (AUC) among the three cells types over the entire assay length (supplementary Fig. 4A). Macrophages were the most efficient phagocytes (AUC: 7000 ± 182.2 NBs co-localized with the phagocytic cells × number of h) followed by OECs (2873 ± 201.3 NBs co-localized with phagocytic cell × h) and then SCs (934.2 ± 55.08 NBs co-localized with phagocytic cell × h).

**Figure 4:**
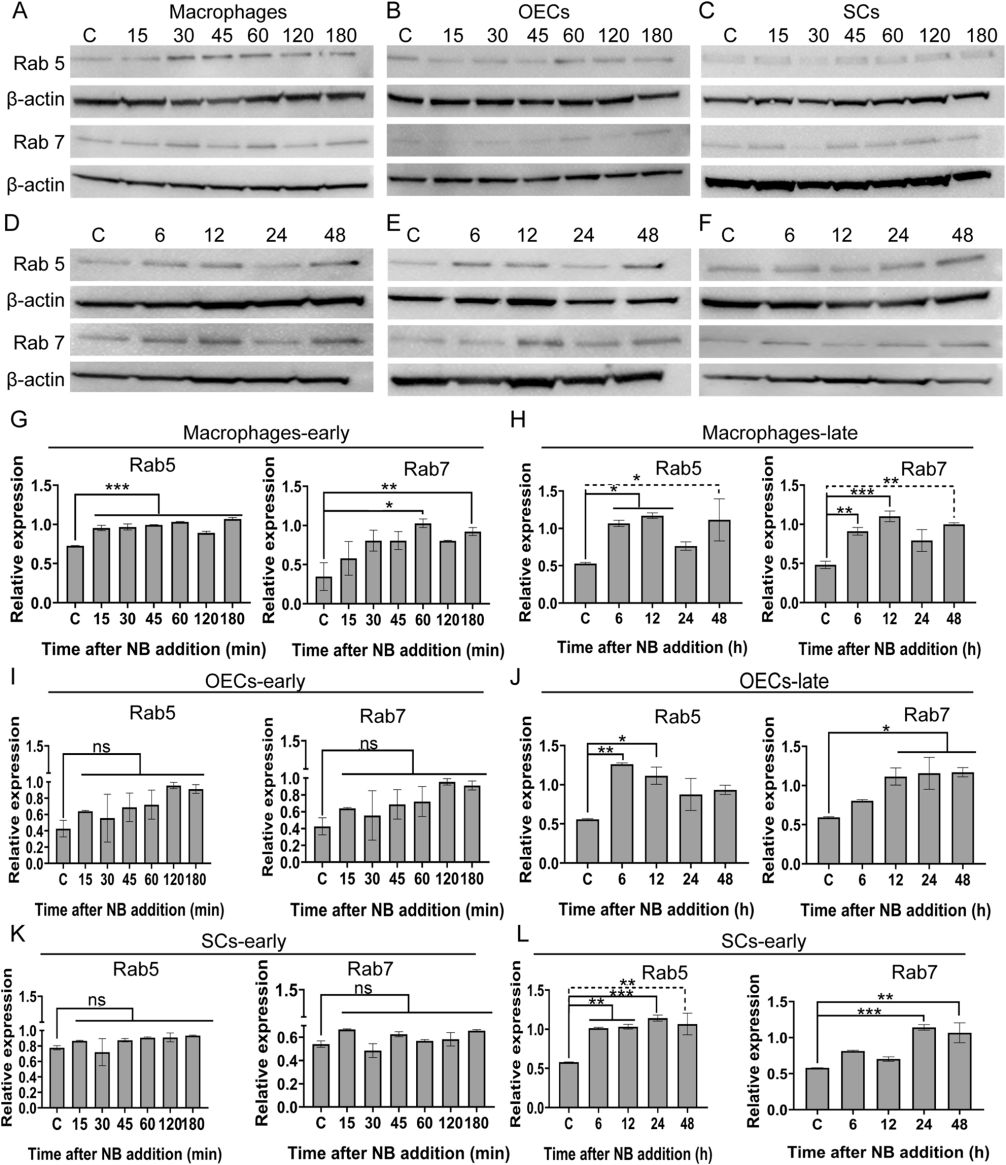
Processing of necrotic bodies in endosomes by macrophages and glia. (**A**–**L**) Expression of early (Rab5) and late (Rab7) endosomal markers in the different cell types after challenge with necrotic cells. (**A**–**F**) Example images of Western Blots for Rab5 and Rab7; (**A**–**C**) show 0–180 min, (**D**–**F**) show 0–48 h. (**G**–**L**) Quantification of Rab5 and Rab7 protein expression. Bar graphs represents relative protein expression of Rab5 and Rab7 to a housekeeping protein (β-actin), with data from three independent experiments ± SEM. *P ≤ 0.05, **P ≤ 0.01, ***P ≤ 0.0001 (one-way ANOVA with Dunnett’s multiple comparison post-hoc test).

When a phagosome fuses with endosome-lysosome, the pH within the vacuole successively decreases. An early phagosome that has merged with an endosome has a pH of around 6.5, a late phagosome that has merged with late endosome has a pH of around 5.5 and a phagolysosome has a pH of around 4.5^43^. The pHrodo dye is fluorescent at all these pHs and thus cannot be used to determine the stage of lysosomal maturation. To investigate the processing of phagocytosed necrotic cargo in the glia, we instead studied the expression of small GTPases of the Rab family, the Ras related proteins 5 and 7 (Rab5 and Rab7, Fig. 4), which are markers of the different stages of endo/lysosomal maturation. Rab5 is required for early phagosome formation, as well as endosomal recruitment and attachment to the phagosome^44,45^. The transition of early to late phagosome is characterized by replacement of Rab5 with Rab7 and the recruitment of late endosomes and lysosomes^46^. Increased Rab 5 expression (in comparison to untreated control cells) was first detected in macrophages at ~ 15 min post NB challenge, whereas Rab 7 expression was first detected at 1 h (Fig. 4A,G). At later time-points, macrophages continued to express Rab 5 and Rab 7 (Fig. 4D,H). In contrast, no altered expression of Rab5 or Rab7 was found in OECs (Fig. 4B,I) and SCs (Fig. 4C,K) up to 3 h post treatment with NBs. Rab 5 expression was detected in glia from 6 h onwards post NB challenge (OEC; (Fig. 4E,J)); (SC; Fig. 4F,L)). In OECs and SCs, up-regulation of Rab 7 expression occurred from 12 h (Fig. 4E,J) and 24 h onwards (Fig. 4F,L), respectively. Full blots of all proteins are shown in supplementary Figure 5 and 6.

**Figure 5:**
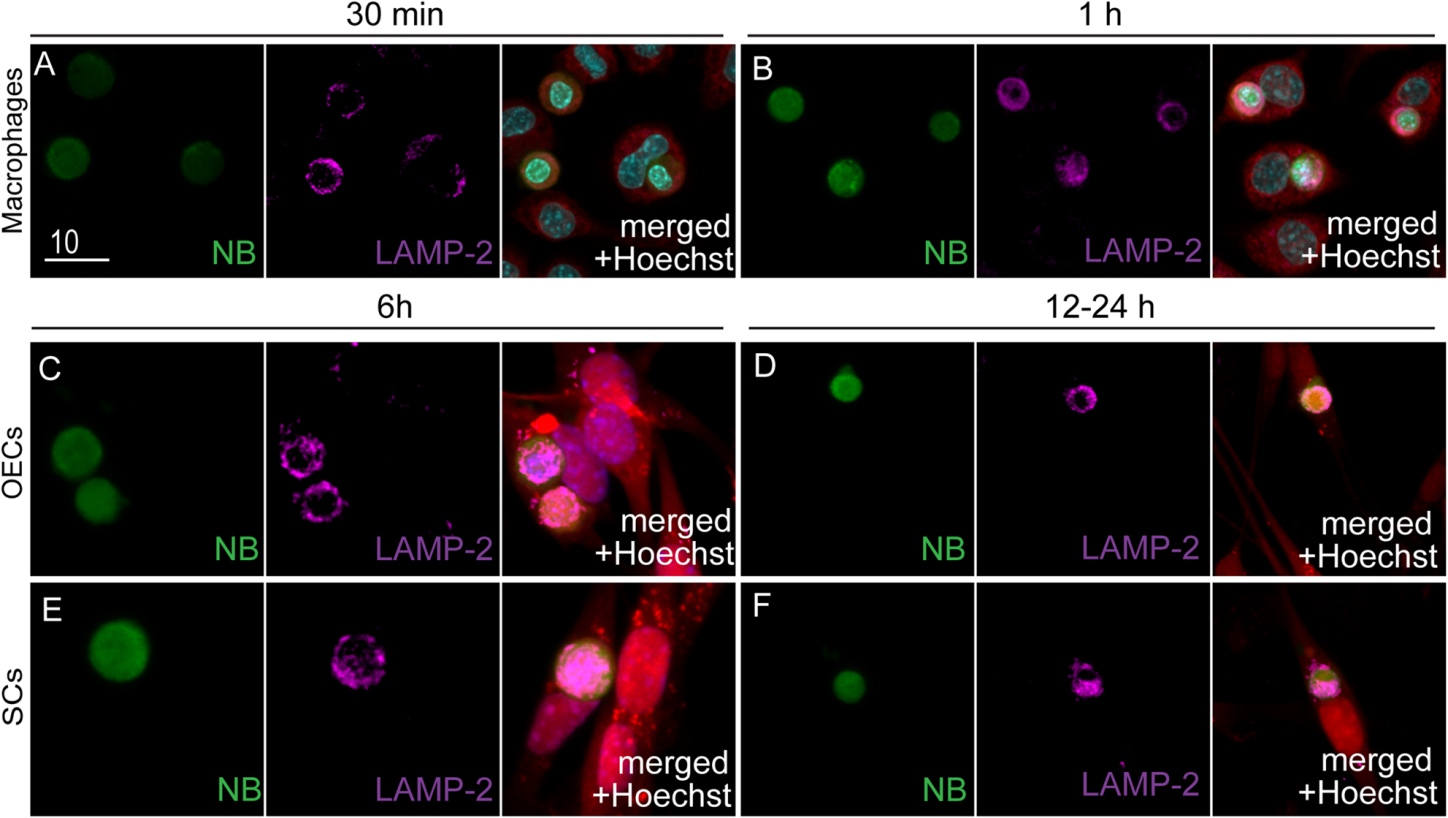
Necrotic bodies co-localize with lysosomes. (**A**–**F**) Co-localisation of internalized NBs with lysosomes (LAMP-2) different time-points post challenge with necrotic cells. Green: internalized NBs. Magenta: LAMP-2 immunolabelling. Red: macrophages (**A**, **B**; CellTracker Red dye) or OECs/SCs (DsRed; **C**–**F**). Blue: nuclear stain (Hoechst). Scale bar: 10 µm.

To further study processing of the necrotic cargo, we also immunolabelled the cells at different time-points post NB challenge for lysosome-associated membrane protein 2 (LAMP-2) (Fig. 5). LAMP-2 s constitute the major protein components of the lysosomal membrane. Their functions include fusion of the lysosomes with phagosomes, as well as acidification of the lysosomal lumen that helps in degradation of the internalized cargo^47^. The presence of a well-defined ring of LAMP-2 around the phagosome is indicative of phagolysosome formation^48^. For macrophages, faint immunolabelling for LAMP-2 was observed around internalized necrotic bodies 30 min after the cells were exposed to NBs (Fig. 5A). After 1 h, clear LAMP-2 labelling was seen surrounding smaller necrotic targets (Fig. 5B). LAMP-2 labelling surrounding internalized NBs was observed at approximately 6 h after NB challenge in both OECs (Fig. 5C) and SCs (Fig. 5E). At ~ 12 h post NB exposure in OECs (Fig. 5D) and after 24 h in SCs (Fig. 5F), smaller necrotic targets surrounded by well-defined LAMP-2-positive lysosomes were seen.

### Glial internalization of necrotic bodies involves phosphatidylserine recognition

**Figure 6:**
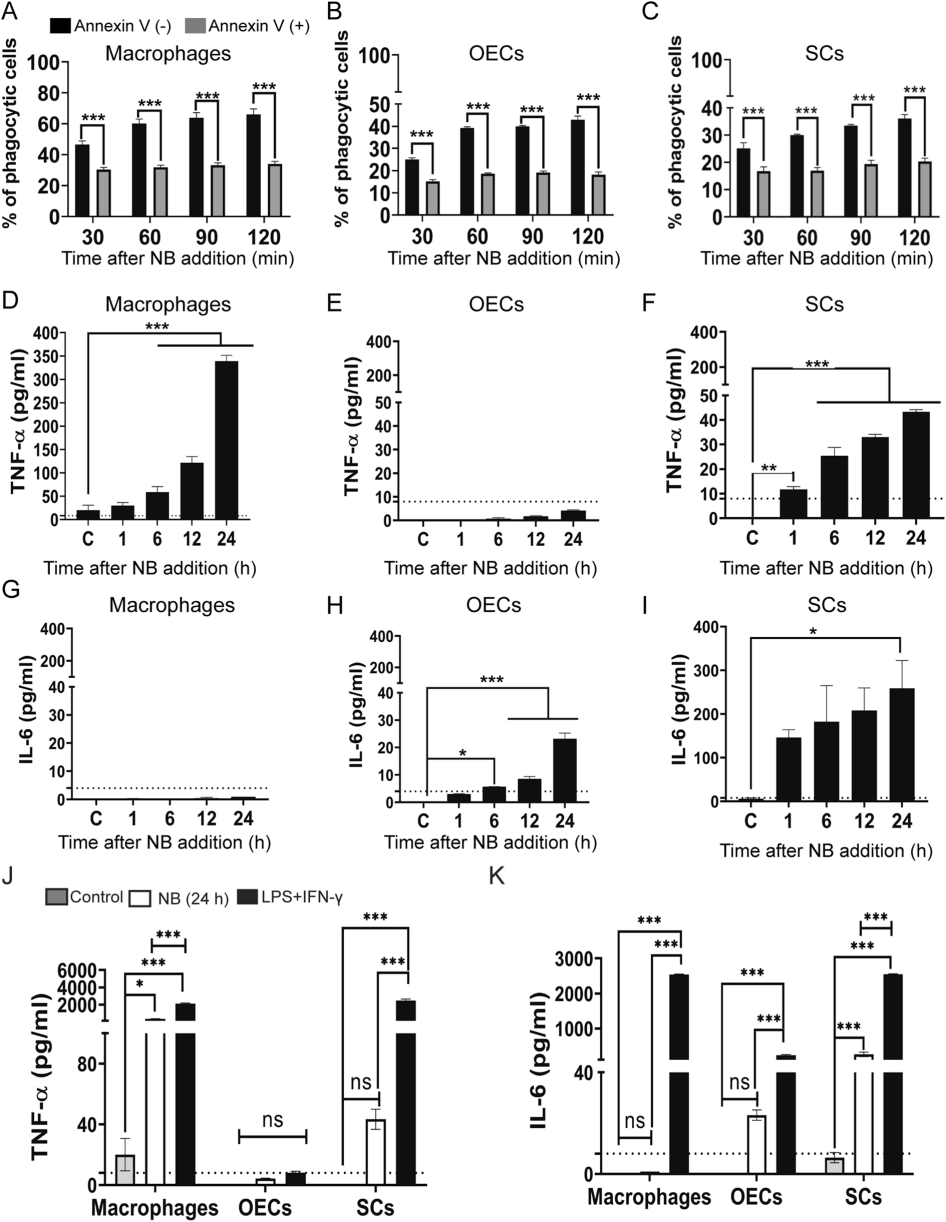
Internalization of necrotic cells by macrophages, OECs and SCs is dependent on PS recognition and results in the production of TNF-α. (**A**–**C**) Blockage of PS by annexin V impairs NB internalization. Macrophages (**A**), OECs (**B**) and SCs (**C**) were exposed to fluorescently labelled necrotic cells (CMFDA dye, green) which had, or had not, been pre-incubated with annexin V. After 2 h, internalization of necrotic debris was determined. ***P ≤ 0.0001 (two-way ANOVA with Sidak’s multiple comparison test). Data represents mean ± SEM. n = 3 biological repeats × 3 technical replicates × 4 FOV (% phagocytic cells: cells with necrotic bodies/total cells × 100). (**D**–**F**) Production of TNF-α (**G**–**H**) Production of IL-6 following exposure to NBs by macrophages, OECs and SCs**.** At different time-point post exposure to NBs, the TNF-α and IL-6 levels produced by the three cell types were measured using ELISA (**D**, **G**: macrophages, **E**, **H**: OECs, **F**, **I**: SCs). **P ≤ 0.01, ***P ≤ 0.0001 (one-way ANOVA, Dunnett’s multiple comparison post-hoc test). Production of TNF-α (**J**) and IL-6 (K) in macrophages, OECS and SCs when exposed to NBs or a strong inflammatory stimulus (LPS + IFN-γ) for 24 h. **P ≤ 0.01, ***P ≤ 0.0001 (one-way ANOVA, Dunnett’s multiple comparison post-hoc test). Detectable TNF-α range of kit was 8–1000 pg/ml; IL-6: 4–500 pg/ml (dotted lines). Data represents three biological replicates with two technical replicates per assay experiments ± SEM.

We then investigated the mechanism by which glia recognize necrotic targets. Both “professional” and “non-professional” phagocytes have been shown to phagocytose necrotic cells by recognition of PS displayed on dying cells^29,30,37,39,49,50^. Hence, prior to exposing macrophages, OECs and SCs to NBs, we incubated the NBs with the PS binding partner Annexin V that binds to PS and blocks PS recognition. Pre-incubation with Annexin V resulted in a significant decrease in the internalization of NBs (~ 40–50%) by all three cell types (Fig. 6A–C).

### Glia respond to necrotic bodies with pro-inflammatory cytokines

The production of both pro- and anti-inflammatory cytokines constitutes an important component of the processing of phagocytosed cargo^42^, but a pro-inflammatory response is detrimental after cell transplantation into a CNS injury site^22,51,52^. We therefore measured the production of pro-inflammatory cytokines (TNF-α, IL-6) by the glia and macrophages in response to NB exposure. We found that macrophages and SCs responded by production of inflammatory cytokines TNF-α, which increased over time with macrophages producing seven times more TNF-α than SCs (Fig. 6D,F). In contrast, OECs did not produce TNF-α above threshold detection levels (Fig. 6E). Surprisingly, macrophages did not produce IL-6 above threshold detection levels post NB exposure (Fig. 6G). In contrast, SCs rapidly produced a large amount of IL-6 (Fig. 6I), while OECs did not produce detectable levels until 6 h after NB exposure and only produced low amounts even at 24 h, with ten times less production than SCs (Fig. 6H). To compare the cytokine response to that of a general pro-inflammatory environment (like the environment in a spinal cord injury site, however, less complex), we also challenged the cells with a combination of two strong pro-inflammatory stimuli, lipopolysaccharide (LPS) and interferon-γ (IFN-γ). Again, both macrophages and SCs produced high levels of TNF-α, while OECs did not produce TNF-α above threshold detection levels. While all three cell types produced significant amounts of IL-6 after exposed to LPS + IFN-γ, OECs produced the least amount of this cytokine (Fig. 6J, K).

### Glia engulf myelin debris that is rapidly processed into acidic endosomal-lysosomal compartments

**Figure 7:**
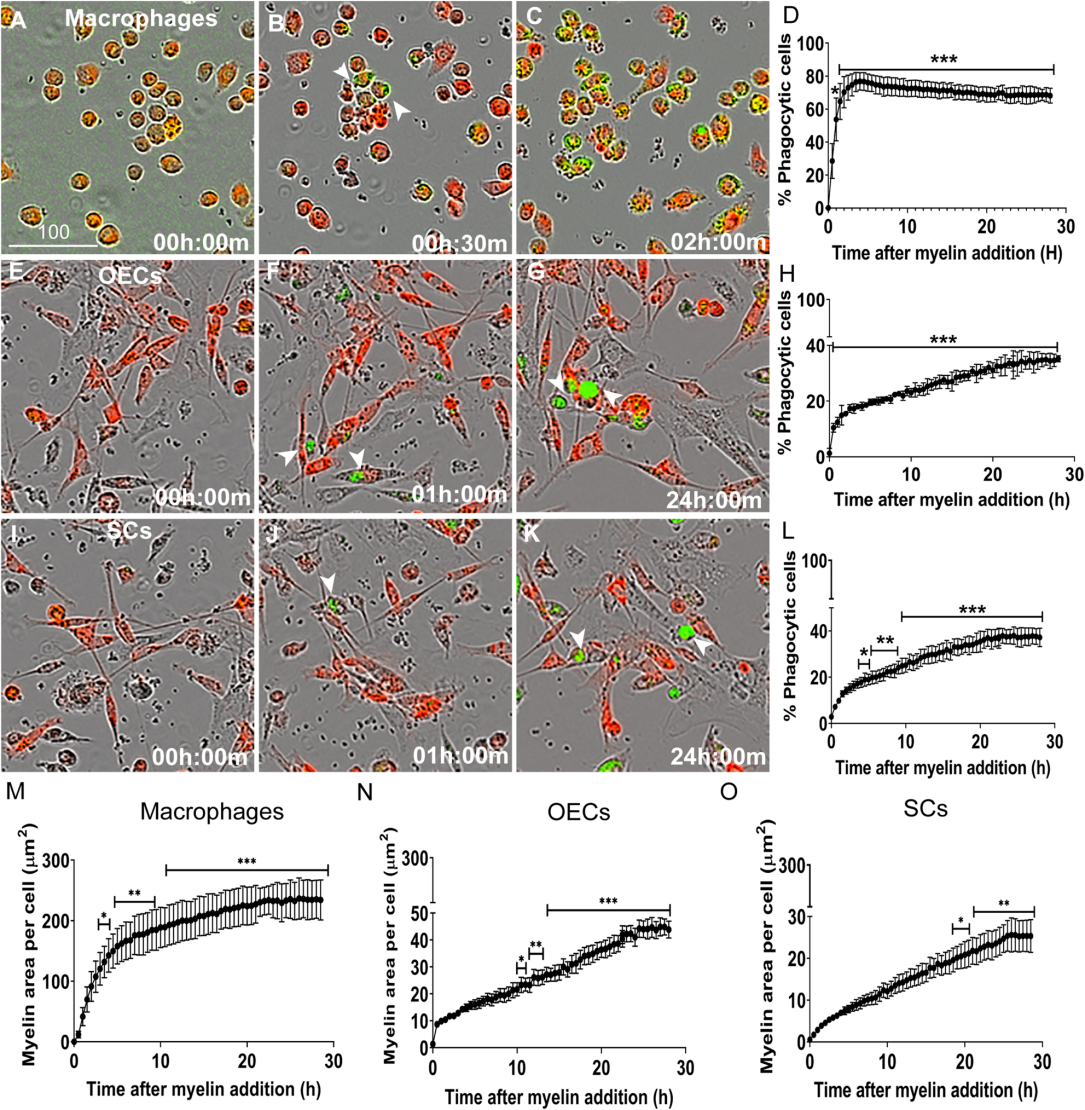
Phagocytosis of myelin by macrophages, OECs and SCs. Shown are example images of macrophages (J774A.1 cells) (**A**–**C**), OECs (**E**–**G**), SCs (**I**–**K**) (red) with myelin debris within endosomes/lysosomes (green, arrowheads). Myelin was labelled with pHrodo STP dye (green) that only fluoresces in acidic compartments (endosomes/lysosomes) within cells. Graphs show the percentage of macrophages (**D**), OECs (**H**) and SCs (**L**) containing myelin debris. Graphs (**M**–**N**) display the area of myelin inside intracellular acidic compartments (green object area) per cell. Asterisks show time-points at which there was a significant difference from background (time zero). *P ≤ 0.05, **P ≤ 0.01, ***P ≤ 0.0001 (two-way ANOVA with Sidak’s multiple comparison test). Data represents mean ± SEM. n = 3 biological repeats × 3 technical replicates × 4 FOV (n = 300–400 cells/FOV).

Accumulated myelin debris has been reported to remain years after CNS injury and is thought to impair regeneration^6^. Thus, the ability to phagocytose myelin is also a highly desirable property of transplanted glia. Hence, we investigated how OECs and SCs responded when challenged with myelin debris, and whether there was any difference in myelin debris phagocytosis between the two glial populations. We extracted myelin from the CNS (mouse brain), which was confirmed to express myelin basic protein (MBP) that could be visualized after internalization by macrophages (supplementary Fig. 7). To compare the phagocytic activity between the two types of glia (and macrophages), the myelin was labelled with pHrodo-STP green dye, thus allowing tracking into endo/lysosomes. The labelled myelin debris was added to cultured macrophages, OECs and SCs, and the phagocytosis assay was conducted similarly to the assay with pHrodo-labelled NBs (see Fig. 3). Fluorescent myelin debris was found in macrophages within 30 min (Fig. 7A–B, arrows), and after 1–2 h, 70–80% of macrophages contained myelin (Fig. 7C). Fluorescent myelin debris was first detected after approximately 1 h in both OECs and SCs (Fig. 7E–G,I–K, arrows). This is in contrast to NBs, which were first detected inside acidic organelles only at 2–4 h post exposure in OECs and at 4–6 h in SCs (see Fig. 3E–L). At ~ 30 h, approximately 30–40% of OECs and SCs, and 70% of macrophages had internalized myelin into acidic organelles (Fig. 7D,H,L).

We also quantified the amount of myelin debris phagocytosed by each cell type by determining the area of pHrodo-mediated fluorescence per cell (green object area per cell; Fig. 7M–O) using automated image analysis. In this assay, the amount of myelin inside cells became statistically significant from background (time zero) in macrophages at 4 h, in OECs at 9.5 h and SCs at 18.5 h post debris exposure. Thus, the results from this assay suggest that OECs process myelin debris faster than SCs. We also compared the capacity for myelin phagocytosis between the three cell types by determining the total amount of myelin debris inside organelles over the entire length of the assay (area under curve (AUC), supplementary Fig. 4B). Macrophages were the most efficient phagocytes, containing the largest amount of phagocytosed myelin debris (AUC: 5399 ± 139 µm^2^ × number of h) amongst the cell types, followed by OECs (777 ± 12 µm^2^ × number of h) and then SCs (445 ± 12 µm^2^ × number of h). The AUC was significantly larger for macrophages than for both glial cell types, and significantly larger for OECs than for SCs. These differences between cell types resembled the differences for phagocytosis of NBs (supplementary Fig.4A,B).

## Discussion

In this study, we demonstrated that peripheral glia (OECs and SCs) phagocytose necrotic bodies (NBs) and process them into phagolysosomes. While OECs and SCs have similar time-courses for the phagocytosis-trafficking pathway, OECs were able to internalize considerably more NBs than SCs and appeared to process them into phagolysosomes at a much greater rate than the SCs. While equal percentages of OECs and SCs were involved in phagocytosis of myelin debris, OECs phagocytosed larger amounts of myelin than SCs. Thus, OECs appear to be more efficient phagocytes than SCs in this model. Whilst the glia internalized necrotic bodies slower than macrophages, the internalization was more rapid than what has previously been reported for “non-professional” phagocytes such as fibroblasts (typically 6–8 h)^50^. Though not investigated in the current study, OECs may also be more efficient phagocytes than astrocytes (“non-professional” phagocytes of the CNS), which have been shown to be incapable of degrading internalized cargo even up to five days post exposure^53^. We also found that OECs produced less pro-inflammatory cytokines than SCs and macrophages, both in response to NBs and in a pro-inflammatory environment.

An important process in phagocytosis is not just engulfment of targets, but also the capacity and efficiency of a phagocyte to degrade the internalized cargo. Prior to the current study, the intracellular fate and time required to process necrotic bodies has not been well characterized for peripheral glia. We found that NBs were trafficked to these compartments in both OECs and SCs, although much slower than in macrophages (2 h to peak in macrophages, 22–23.5 h for SCs and OECs). We also determined that expression of protein markers of early (Rab5)^44,45^ and late phagosomes (Rab7)^54^, as well as for phagolysosome formation and degradation of cargo (LAMP-2)^48^ were up-regulated by both OECs and SCs after internalization of NBs. We observed subtle differences in the efficiency of clearing necrotic bodies between the two glial populations, with OECs appearing to be more efficient at degrading NBs than SCs.

We verified that glial recognition of necrotic bodies occurred mainly via binding to phosphatidylserine (PS); competitive blocking of PS by Annexin V conjugate resulted in decreased NB uptake by 40–50% in all cell types, which is similar to previous reported data for macrophage internalization of cargo^30,37,39^. However, the molecular alterations at the plasma membrane by necrotic cells remain to be characterised. Along with PS, NBs may also display other surface molecules that can be recognized by phagocytes, such as Annexin 1 and calreticulin, which work in conjunction with PS^28^. Necrotic cells can also display DAMPs, which may contribute to their recognition by phagocytic macrophages and glia^55^. Thus, while Annexin V conjugate blocking of PS was effective in reducing phagocytosis, other recognition molecules are likely to also be involved.

Along with necrotic cell-derived debris, myelin debris is also generated following nervous system injury. Accumulation of myelin at the site of injury is thought to increase inflammation and impairment of regeneration^56,57^. Therefore, we also investigated the ability of peripheral glia to phagocytose myelin debris. Engulfed myelin debris was rapidly processed into endosome/lysosomes by both OECs and SCs. We observed some differences between the two glial populations in their phagocytosis of myelin, with OECs phagocytosing myelin faster than SCs. Also, OECs were capable of overall phagocytosing more myelin debris than SCs.

Within the injured spinal cord and after traumatic brain injury, TNF-α and IL-6 are the two major cytokines produced by peripheral immune cells and activated glia adding to detrimental neuroinflammation^1,2^. We therefore focussed on determining whether OECs and SCs produced these cytokines in response to NBs, as well as to a pro-inflammatory stimulus; we also compared cytokine secretion by glia and macrophages. OECs did not secrete detectable levels of TNF-α, neither in response to NBs nor within a pro-inflammatory environment. In contrast, SCs secreted TNF-α in both these conditions. Both OECs and SCs produced IL-6 when challenged with NBs and under pro-inflammatory stimulation, however, OECs consistently produced lower levels of this cytokine than SCs. In fact, in the pro-inflammatory milieu, the production of both TNF-α and IL-6 by SCs was similar to that of macrophages. Studies regarding the macrophage response after phagocytosis of necrotic cells have been conflicting. While some studies have shown that macrophages produce a range of pro-inflammatory cytokines and chemokines such as TNF-α, IL-6 and MCP-3^28,49,58^, we here observed that macrophages produced a large amount of TNF-α production post challenge with NBs, and continued to do so up to 24 h post-challenge (the latest time-point investigated). Surprisingly, in the current study, macrophages did not produce detectable levels of IL-6 after NB challenge, and only produced this cytokine in a pro-inflammatory environment (LPS + IFN-γ).

The strong capacity for phagocytosis is consistent with the role of OECs within the olfactory nervous system. Due to the direct exposure of olfactory sensory neurons to the environment within the nasal cavity, the neurons have a limited lifespan and are constantly replaced with 1–3% of neurons dying each day^59^. The axons of these dead neurons must therefore be constantly removed and OECs are the key phagocytes clearing the resulting debris^10,11^. As the turnover of neurons is a normal and continuous process, it is important that an inflammatory environment is not created by the OECs that would otherwise comprise the functioning of the olfactory nerve. Similarly, recruitment of macrophages is not desirable as it would require continual infiltration of macrophages into the olfactory nerve^13,60^. Indeed, rather than attracting macrophages, OECs appear to repel these cells; MIF secretion from both OECs and macrophages have been shown important for this segregation^12^.

In contrast to the olfactory nerve, other peripheral nerves do not continuously regenerate but only do so after injury, after which SCs are capable of phagocytosing cell debris. When an injury to peripheral nerves does occur, SCs initiate the primary phagocytic response, but also release pro-inflammatory cytokines that attract macrophages to the site, aiding in the clearing of debris and dead cells^13^. Previous studies have shown that SCs produce high IL-6 levels after peripheral nerve injury, which is essential for recruitment of macrophages to clear the majority of debris and dead cells^13,61^. Thus, the difference between OECs and SCs appears to be that (1) there is a lower overall requirement for SCs than for OECs to be continuously phagocytic and (2) OECs secrete less macrophage-attracting signals than SCs^13^. The results from the current study are in accordance with this theory, demonstrating that OECs have a higher capacity for phagocytosis of both NBs and myelin than SCs, and OECs also produce less pro-inflammatory cytokines than SCs during this process.

### Conclusion

Transplantation of glia, in particular OECs and SCs, is being trialled for repair of CNS injuries. One key function of these glia is to remove dead cells, in particular necrotic bodies (NBs), as well as clearing myelin debris. To improve the therapeutic potential of glial transplantation, it is crucial to determine which of the two cell types has the strongest capacity for phagocytosis, and which of the cells secretes the highest levels of pro-inflammatory cytokines that are detrimental to the injury site. It is also important to determine the cellular mechanisms by which the glia respond to NBs, to potentially identify novel drug targets that can be used to stimulate phagocytosis. In the current study, we showed that both OECs and SCs are capable of phagocytosing and trafficking NBs, but that OECs are more effective than SCs in this process. We showed that both glial cells can phagocytose myelin debris, again with OECs being more effective than SCs. We also showed that OECs produce less pro-inflammatory cytokines than SCs. Therefore, transplantation of OECs may be more favourable than transplantation of SCs for therapeutic repair of nervous system injuries.

## Materials and methods

### Cell culture

Primary cultures of glia were prepared from S100β-DsRed transgenic mice as previously described^36,41,62^. Briefly, postnatal day 7 pups were decapitated followed by tissue dissection. For culture of OECs, the nerve fibre layer of the olfactory bulb was isolated and for SC culture, the trigeminal ganglia were isolated. The explants were transferred with glia culture medium to a 24-well plate pre-coated with matrigel (BD Bioscience, diluted 1:10 in DMEM). Glial medium consisted of Dulbecco’s Modified Eagle Medium (DMEM) (Gibco) with 10% fetal bovine serum (FBS), gentamycin at 50 μg/ml (Gibco), l-glutamine at 200 μM (Gibco) and G5 supplement (Gibco). After glial cells emerged from the explants and reached confluence, they were plated for assays. Approximately 80% DsRed positive cells were obtained through this method which we use routinely, and we have previously shown that these DsRed-positive cells are also positive for glial markers (the p75 neurotrophin receptor, s100)^36,41,62^. The McCoy B cell line (ATCC, CRL-1696) was maintained in DMEM supplemented with 10% FBS, l-glutamine at 200 μM and gentamycin at 50 μg/ml). The mouse macrophage J774A.1 cell line (ATCC, TIB-67) was maintained in DMEM medium supplemented with 10% FBS, l-glutamine (200 μM) and gentamycin (50 μg/ml).

All experiments containing animals and transgenically modified cells were conducted with the approval of the Griffith University Biosafety Committee (NLRD/09/15_var7) and the Griffith University Animal Ethics Committee (MSC/13/18/AEC) in accordance with guidelines of the Australian Commonwealth Office of Gene Technology Regulator and the National Health and Medical Research Council of Australia.

### Induction of necrosis

To induce necrosis, McCoy B cells were suspended at 1 × 10^6^ cell/ml in culture medium and heated at 55 °C for 30 min. To confirm cell death and display of phosphatidylserine (PS), an apoptosis/necrosis kit (Abcam) was used as per the manufacturer’s protocol. Heat-treated and control (live) cells were incubated with CytoCalcein Violet (EX/EM: 405/450 nm), membrane-impermeable DNA Nuclear Green DCS1 dye (Em/Ex: 490/520 nm) and the PS sensor dye Apopxin Deep Red (Em/Ex: 630/660 nm), and imaged using a Nikon-Ti-2 epifluorescence microscope. Cells with permeabilized membranes (DCS1 positive) and cells displaying PS (Apopxin-positive) were quantified using NIS Elements General Analysis software.

### Extraction of myelin

Myelin was extracted from the CNS (brain) as previously described^63^. Briefly, brains were dissected from 10–12 S100β-DsRed transgenic mice (12 months old) and transferred to 0.32 M sucrose solution in which they were homogenized. The homogenate was layered onto 0.83 M sucrose and centrifuged at 100,000×*g* for 45 min at 4 °C using an ultracentrifuge. Crude myelin debris was collected from the interface of the two sucrose densities and resuspended in Tris–Cl buffer (1 M Tris.Cl, 2 mM Na_2_EDTA, pH 7.45) following another round of homogenization. The homogenate was centrifuged twice at 100,000×*g* for 45 min at 4 °C; each time, the supernatant was discarded and the white myelin pellet was collected. This myelin pellet was resuspended in sterile PBS and centrifuged at 22,000×*g* for 10 min at 4 °C. The myelin pellet was weighed and stored at a concentration of 50 mg/ml at − 80 °C.

### Phagocytosis assay

Host cells (OECs, SCs and J774A.1 macrophages) were seeded at a density of 6000 cells per well in a 96-well plastic plate. OECs and SCs express DsRed fluorescent protein; macrophages were labelled with CellTracker Red CMPTX Dye (ThermoFisher), allowing visualization of cells in the red channel. For the necrotic body (NB) internalization assay, NBs were labelled with Celltracker Green CMFDA Dye (ThermoFisher) prior to induction of necrosis as described above. To visualise NB entry into endosomes/lysosomes, NBs were labelled with pHrodo Green STP Ester dye (pHrodo STP; ThermoFisher) post induction of necrosis as per the manufacturer’s guidelines. In brief, NBs were washed twice with PBS and resuspended at 1 × 10^6^ cells/ml in 0.1 M sodium bicarbonate buffer at pH 8.4, containing 5 µm pHrodo STP, and incubated for 1 h at room temperature. NBs were then washed twice in PBS and resuspended in OPTI-MEM medium. For PS-blocking experiments, NBs were collected, washed in cold PBS and resuspended in annexin binding buffer (10 mM HEPES, 140 mM NaCl and 2.5 mM CaCl_2_, pH 7.4) with Annexin V Alexa Fluor 647 conjugate (ThermoFisher) (5 µl/100 µl assay) for 15 min at room temperature. For all phagocytosis assays, NBs were added to host cells in OPTI-MEM medium (ThermoFisher) at a ratio of 4:1, while myelin debris was added at 1 mg/ml, and imaged every 30 min using an IncuCyte live cell imaging system (10 × objective and 30-min imaging intervals) capturing 4 fields of view (FOV) per well. To quantify internalization of NBs, the number of NBs co-localizing with cells was determined, indicating that the cells had engulfed the NBs; area under the curve (AUC) was calculated to determine the number of NB co-localisations over time. OECs and SCs were visualised by expression of the fluorescent protein DsRed, macrophages were visualised with CellTracker Red dye, and NBs were tagged either with CellTracker CMFDA dye or pHrodo STP (both green). Images were analysed using Cell Profiler software (cellprofiler.org) as previously described^18^. To verify NBs were internalized by the cells and not merely attached to the membrane, after 2 h of addition, excess NBs were washed off in cold PBS, followed by fixation in 4% paraformaldehyde (PFA) and imaging using confocal microscopy. We then performed 3D rendering using Imaris 7.4.2 software to determine whether NBs were present inside cells.

For myelin phagocytosis assays, the brain-derived myelin debris was labelled with pHrodo Green STP Ester dye (pHrodo STP; ThermoFisher). Myelin debris was resuspended at 5 mg/ml in 0.1 M sodium bicarbonate buffer at pH 8.4, containing 12.5 µM pHrodo-STP and incubated for 1 h at room temperature on a shaker, facilitating gentle agitation. After pHrodo labelling, the myelin was then washed thrice in PBS. Myelin phagocytosis assays were conducted according to the same protocol as the assays assessing internalization of NBs into endo/lysosomes. However, while NBs consist of intact cells, myelin consists of debris/very small particles. Hence, to quantify myelin phagocytosis, the area of engulfed myelin debris displaying green fluorescence (thus being inside acidic intracellular compartments) per cell was calculated using Cell Profiler software (cellprofiler.org) as previously described^18^ and represented as “green fluorescent object area”.

### Immunofluorescence

Cells were fixed with 4% PFA in PBS for 15 min, then blocked and permeabilized with 0.3% Triton-X in bovine serum albumin (BSA, 3%) for 15 min. For LAMP-2 immunolabelling, cells were permeabilized with 0.01% saponin as per manufacturer’s instructions, followed by blocking with 3% BSA for 30 min. Cells were incubated with the following primary antibodies overnight at 4 °C; anti-p75ntr (4 µg/ml; BioLegend); anti-S100β (4 µg/ml; ThermoFisher) and anti-LAMP-2 (4.2 µg/ml; Abcam), anti-MBP (4.3 µg/ml; Gene Tex) The following day, cells were incubated with secondary antibodies donkey anti-rabbit IgG Alexa Fluor 488 (4 µg/ml; Abcam), donkey anti-rat IgG Alexa Fluor 647 (4 µg/ml; Abcam) or donkey anti-rabbit IgG Alexa Fluor 647 (4 µg/ml; Abcam; ThermoFisher). Nuclei were stained using Hoechst 33,342 (1 μg/ml, ThermoFisher). High resolution images were obtained using a confocal microscope (Olympus FV3000).

### Western Blot

OECs, SCs and macrophages were seeded onto 6-well plates at 8 × 10^5^ cells per well. The following day necrotic cells were added (at 4:1 ratio) to cells. The plates also contained control cells without NBs. At different time-points (15 min, 30 min, 45 min, 1 h, 2 h, 3 h, 6 h, 12 h, 24 h, and 48 h) cells were washed with ice-cold PBS three times to remove all floating NBs, followed by collection of cells using cell scrapers. After another wash with PBS, cells were resuspended in cold radioimmunoprecipitation assay (RIPA) lysis and extraction buffer (ThermoFisher) supplemented with Halt protease inhibitor cocktail diluted as per manufacturer’s instructions (ThermoFisher). After 15 min, the cell lysates were centrifuged for 20 min at 15,000×*g* at 4 °C. The supernatant was collected, and the protein concentration of total cell lysates was determined by using the Pierce BCA Protein Assay Kit (ThermoFisher). Equal amount of proteins (20 µg) were separated on a 10% Bolt Bis–Tris Plus pre-made gel (ThermoFisher) and transferred to an iBlot 2 PVDF mini stack membrane (ThermoFisher). The membranes were placed in blocking solution (5% skim milk powder in PBS-Tween (0.1% Tween20; PBS-T) buffer for 1 h at room temperature. The membranes were incubated with primary rabbit antibodies against Rab 5 (1:1000, Abcam) or Rab 7 (1:1000) overnight at 4 °C. For comparison of protein expression, the housekeeping protein β-actin was detected using a primary mouse antibody (1:2000, Abcam). This was followed by incubation with secondary anti-rabbit and anti-mouse antibodies conjugated with horseradish peroxidase (HRP 1:2000, Abcam) for 1 h at room temperature. Immobilon western chemiluminescent HRP substrate (Merck Millipore) was used to detect antigen–antibody reaction, followed by exposure and imaging of the membranes using a Chemidoc MP (Bio-Rad) imaging station. The levels of the Rab 5 and Rab 7 proteins relative to β-actin were quantified by densiometry analysis using Image J (version 1.52b).

### Cytokine analysis

OECs, SCs, macrophages were plated onto 24-well plastic plates at a density of 1.25 × 10^5^ cells/well. When cells were 70–80% confluent, NBs were added (4:1 ratio as before) and supernatants were collected after 1 h, 6 h, 12 h, and 24 h. Supernatants were also collected from unchallenged cells (negative control) and cells challenged with inflammatory stimulus; interferon-γ (IFN-γ) 10 ng/ml (Abcam) + lipopolysaccharide (LPS) from *E. coli* (100 ng/ml) (Sigma) for 24 h. Supernatants were stored at – 80 °C. As IL-6 and TNF-α production in cells were high, particularly with inflammatory challenge, experiments contained both neat and samples diluted with ELISPOT diluent (1:10) to ensure readings were within detectable range of the kit. TNF-α and IL-6 levels were measured using sandwich ELISA as per manufacturer’s instruction (ThermoFisher). Endpoint reading was performed measuring absorbance at two wavelengths: 450 nm and 570 nm and the resultant absorbance values were obtained by subtracting 570 nm reading from 450 nm. Absorbance was measured using POLARstar® Omega plate reader (BMG Labtech).

### Software and statistical analyses

Cell Profiler 3.19 software (cellprofiler.org) was used throughout for image analyses. Images were colour-balanced uniformly across the field of view using Adobe Photoshop Creative Cloud 2018 (19.1.4) and compiled into panels using Adobe Illustrator Creative Cloud 2018 (22.1). GraphPad Prism 8 Software was used for statistical analyses. Statistical tests used included one-way ANOVA, two-way ANOVA with Dunnett’s or Sidak’s multiple comparison post-hoc test and unpaired t-test with Welch’s correction.

## Supplementary information

Supplementary Figures.

Supplementary Video 1.

Supplementary Video 2.

Supplementary Video 3.
