# Supplementary material for: Key differences between olfactory ensheathing cells and Schwann cells regarding phagocytosis of necrotic cells: implications for transplantation therapies
Source: Sci Rep. 2020 Nov 3;10:18936. doi: 10.1038/s41598-020-75850-8 (PMC7642263; doi:10.1038/s41598-020-75850-8)
Supplement: Supplementary file 1 — Supplementary Figures. [file 41598_2020_75850_MOESM1_ESM.docx]

**­­Key differences between olfactory ensheathing cells and Schwann cells regarding phagocytosis of necrotic cells – implications for transplantation therapies**

Nazareth L, Shelper TB, Chacko A, Basu S, Delbaz A, Lee JYP, Chen M, St John JA, Ekberg JAK


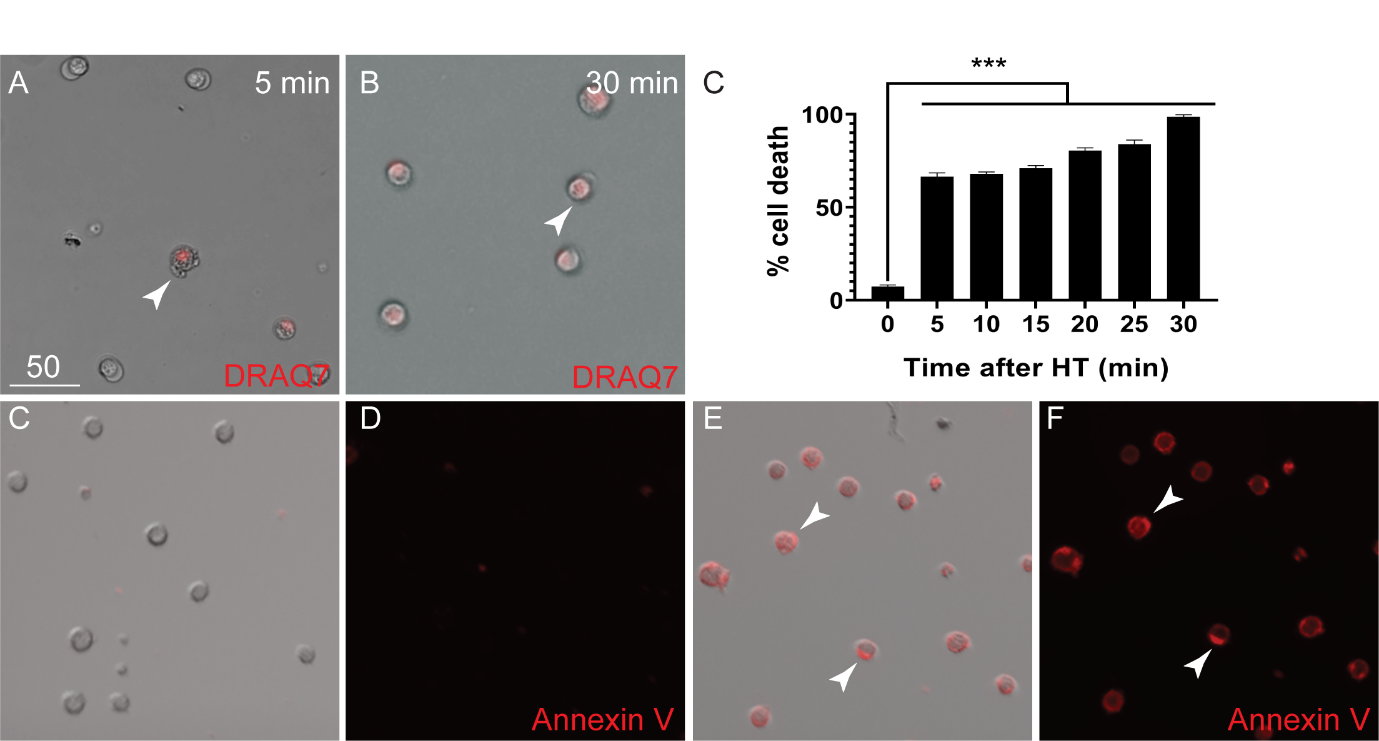


**Supplementary Figure 1.** Heat-treated (HT) McCoy B cells become necrotic and display PS. (A-B) Representative images of McCoy B cells exposed to 55.4 °C heat treatment and labelled with DRAQ7 (red) after (A) 5 min or (B) 30 min of heat treatment. (C) Graphical representation of % cell death post HT. (C-F) Control cells (C, D) and HT-treated cells (E, F) labelled with fluorescent Annexin V Alexa 647 conjugate (red). Scale bar: 50 µm.


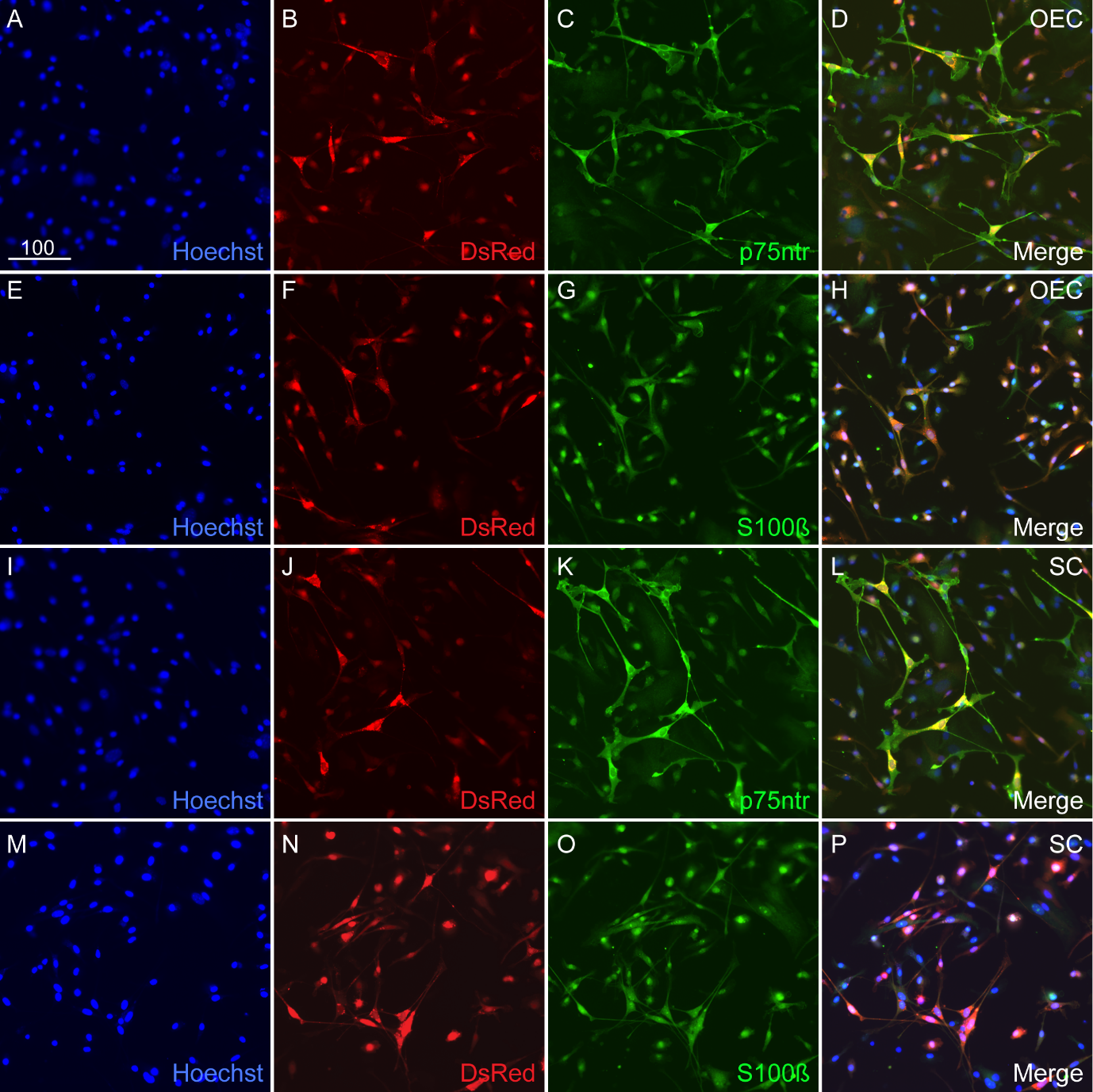


**Supplementary Figure 2.** OECs and SCs express the glial markers p75NTR and S100-β. (A-D), I-L) OECs and SCs immunolabelled for p75NTR. (E-H; M-P) OECs and SCs immunolabelled for S100-β. Blue: nuclear dye (Hoechst), red: glial cells (DsRed), green: immunolabelling for p75NTR (C, K) or S100-β (G, O); (D, H, L, P) merged images of their respective row. Scale bar: 100 µm.


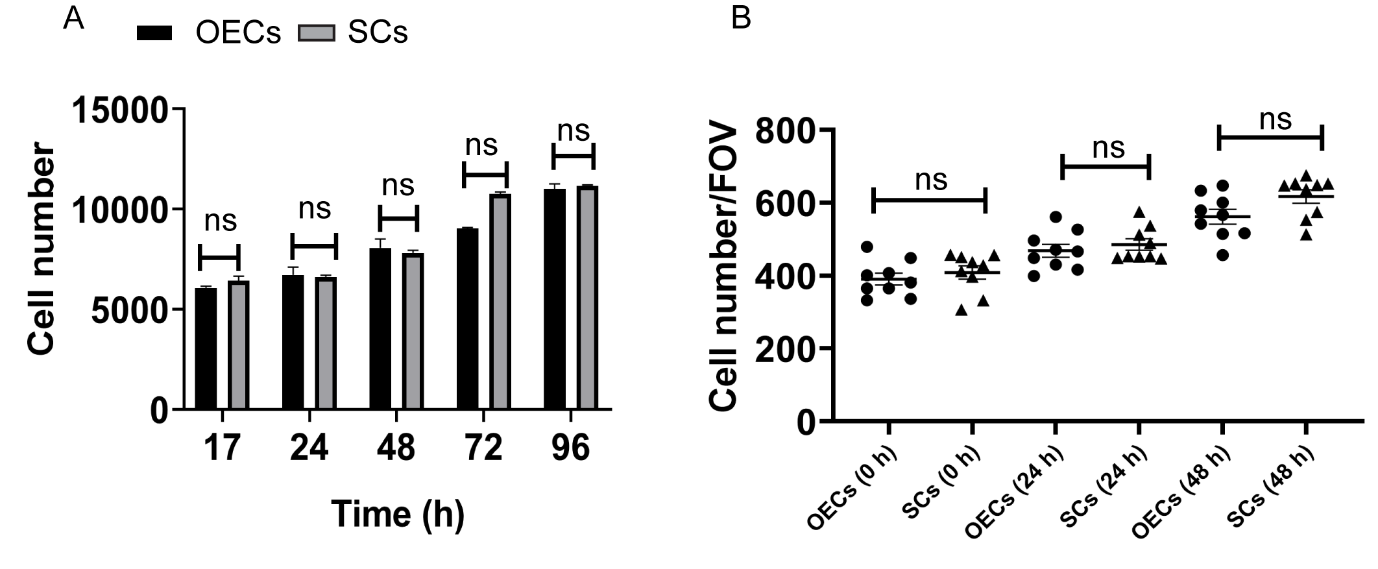


**Supplementary Figure 3.** OECs and SCs have similar proliferation rates. (A) Graphical representation of doubling time for OECs and SCs over 96 h, initial seeding density for both cells was 6,000 cells in 96-well plate. (B) OEC (circles) and SC (triangles) numbers during phagocytosis assays calculated per FOV. One-way AOVA, Sidak’s multiple comparison test.


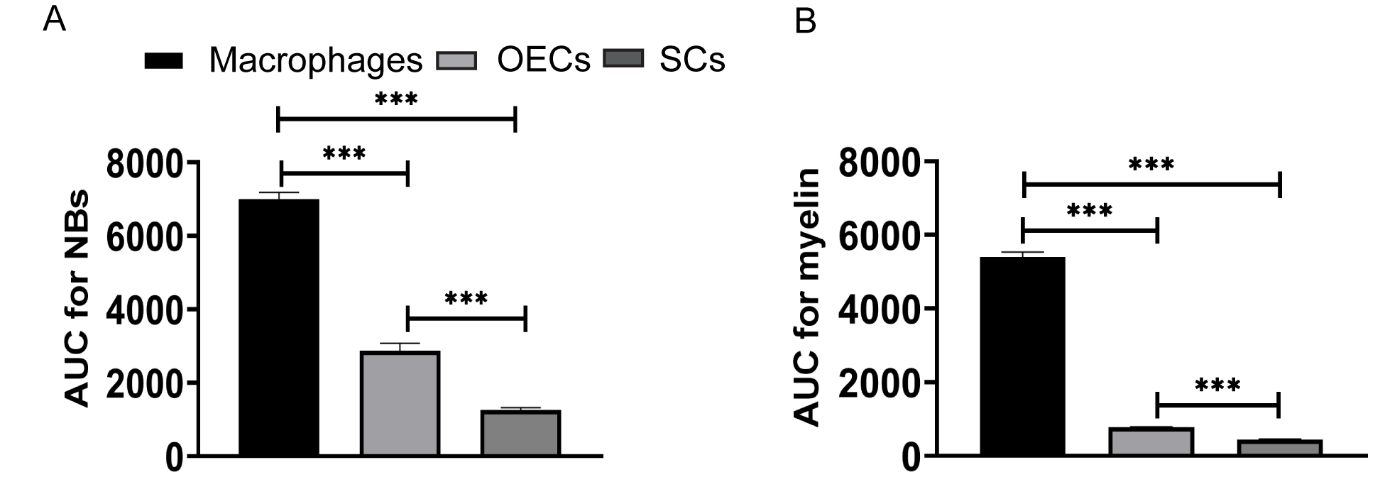


**Supplementary Figure 4.** Comparison of glial/macrophage-mediated internalization of necrotic bodies and myelin debris. Cells were exposed to pHrodo-tagged (A) NBs and (B) myelin debris and amount of fluorescent material inside cells quantified. For NBs, number of fluorescent objects co-localizing with cells determined (number of green objects co-localizing with cells) over time (30 h). For myelin debris, which is composed of multiple small irregular size objects, the area occupied by green fluorescence per cell was determined (green object area/cell), also over 30h. To estimate overall capacity of phagocytosis for each cell type, area under curve (AUC) over the entire duration of assay (30 h) was calculated. ***P≤0.001 (one-way ANOVA, Tukey’s multiple comparison post-hoc test).


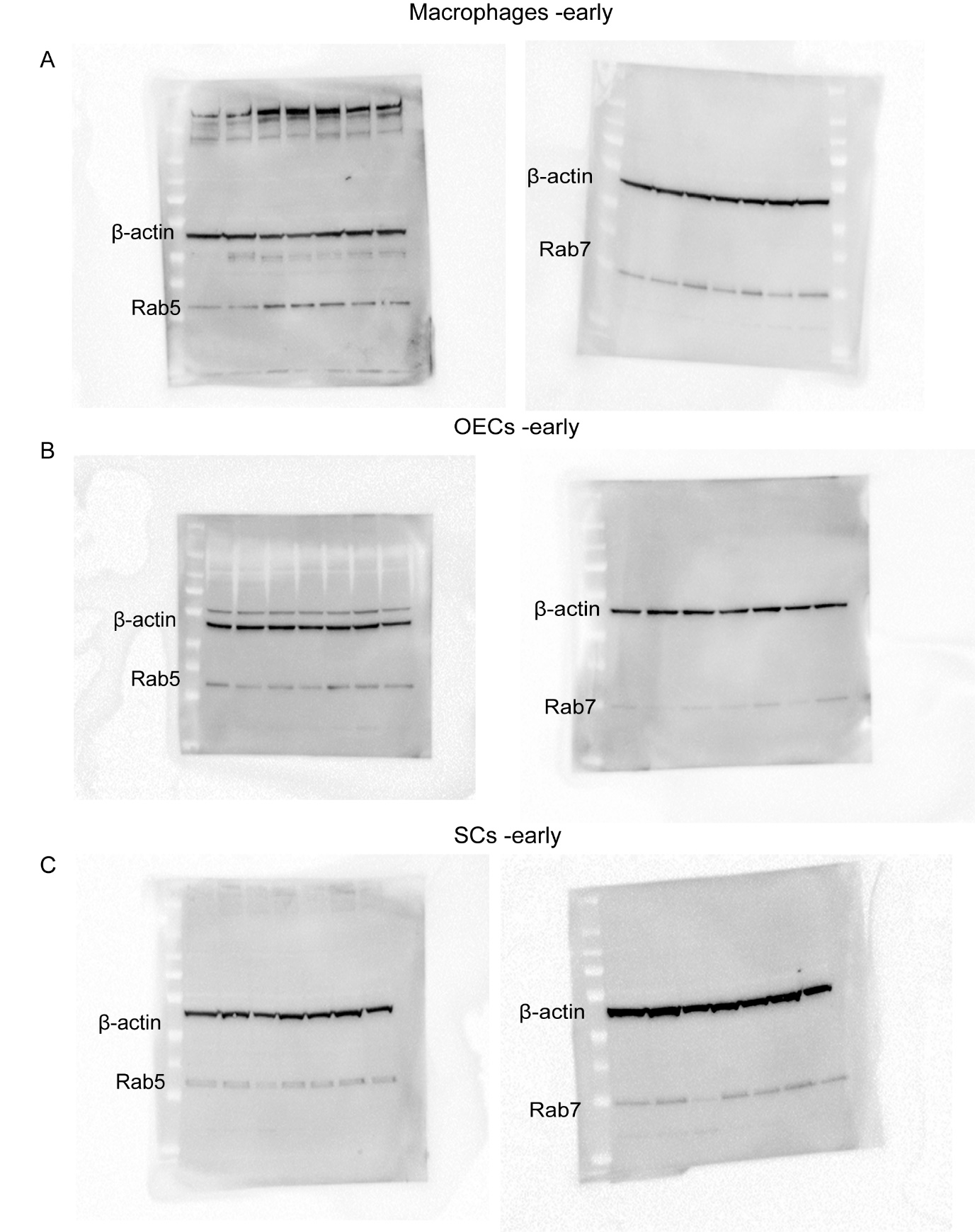


**Supplementary Figure 5.** Uncropped Western Blot images of Rab5 and Rab7 (0-180 min): A-C: Western Blots for Rab5 and Rab7 expression of (A) macrophages, (B) OECs and (C) SCs challenged with NBs for 0-180 min.


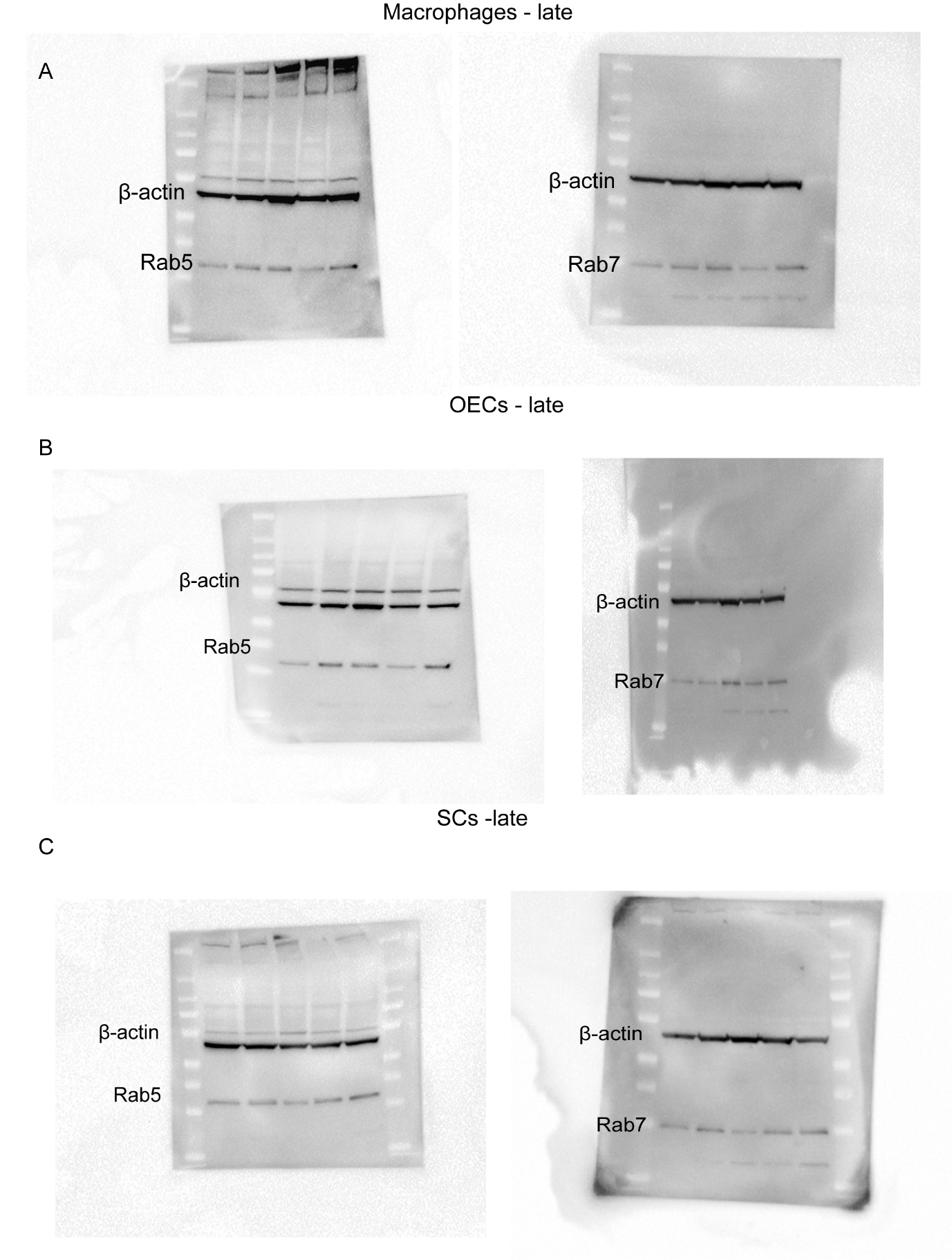


**Supplementary Figure 6.** Uncropped Western Blot images of Rab5 and Rab7 (0-48 h): A-C: Western Blots for Rab5 and Rab7 expression of (A) macrophages, (B) OECs and (C) SCs challenged with NBs for 0-48 h min.


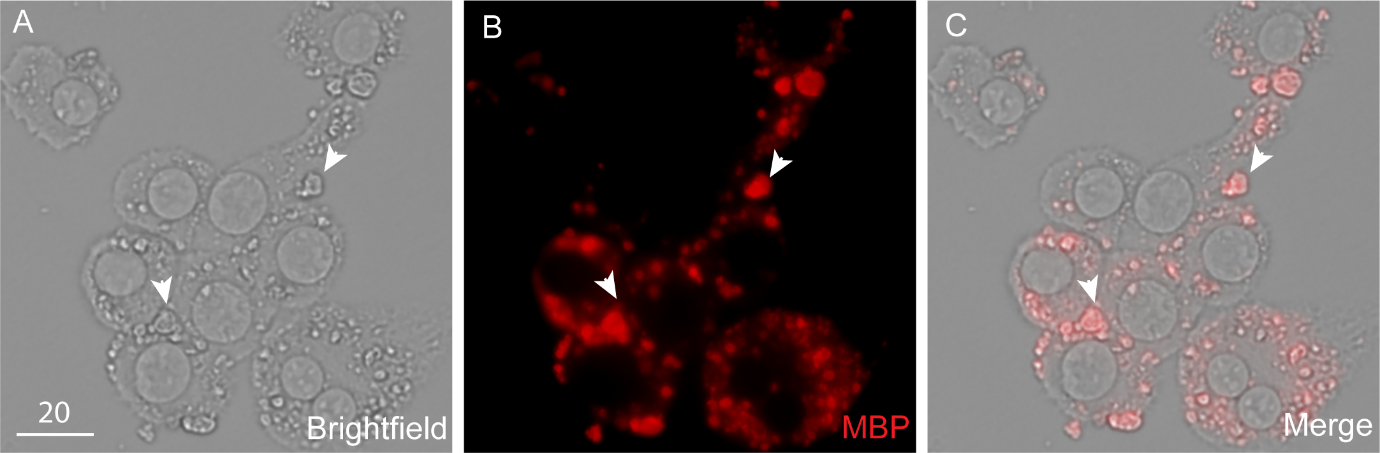


**Supplementary Figure 7.** Internalization of myelin debris by macrophages. Cultured macrophages were challenged with myelin debris. After 2 h, cells were fixed and immunolabelled for myelin basic protein (MBP). (A) brightfield image, (B) MBP immunolabelling (red fluorescence), (C) merged images of A-B. Scale bar: 20 µm.

**Supplementary video 1.** 3D rendered confocal image of macrophages (red) with internalized NB (green), 2 h after addition. Macrophages (CellTracker red dye), NB (CMFDA green dye), Nucleus (Hoechst, blue).

**Supplementary video 2.** 3D rendered confocal image of OECs (red) with internalized NB (green), 2 h after addition. OECs (DsRed), NB (CMFDA green dye), Nucleus (Hoechst, blue).

**Supplementary video 3.** 3D rendered confocal image of SCs (red) with internalized NB (green), 2 h after addition. SCs (DsRed), NB (CMFDA green dye), Nucleus (Hoechst, blue).
